# A Conceptual Framework for Understanding Patient Expectations in Individualised Anaesthesia and Analgesia: A Narrative Review and Future Directions

**DOI:** 10.3390/jpm16040191

**Published:** 2026-04-01

**Authors:** Krister Mogianos, Anna K. M. Persson

**Affiliations:** 1 Department of Anesthesiology and Intensive Care Medicine, Halland Hospital, 301 85 Halmstad, Sweden; 2 Department of Clinical Sciences Malmo, Lund University, 202 13 Malmo, Sweden

**Keywords:** Bayesian modelling, prior expectations, predictive coding, postoperative pain, individualised anaesthesia

## Abstract

Acute postoperative pain remains a major clinical challenge, affecting both recovery and resource utilisation. Beyond nociceptive input, pain is shaped by cognitive and emotional factors, including patient expectations. This narrative review examines the role of expectations in perioperative pain modulation, framed within predictive coding and Bayesian inference models. These models conceptualise pain as a probabilistic process that integrates sensory input with prior expectations, weighted by precision. In theory, positive expectations may enhance analgesic efficacy, whereas negative expectations may amplify pain via nocebo mechanisms. Control modifies expectations and may reduce perceived pain, while uncertainty diminishes these benefits. Evidence from observational studies links preoperative pain self-efficacy and anticipated pain scores to postoperative outcomes, yet interventional trials remain scarce. In this narrative review, we propose that expectation-sensitive strategies, including structured communication and computational modelling, may inform individualised anaesthesia and analgesia. Future research should validate these frameworks in clinical trials, optimise preoperative expectation management, and explore synergistic approaches that combine pharmacology with cognitive modulation. Understanding and leveraging expectations may offer a promising conceptual direction for more individualised perioperative care, although this approach remains hypothesis-generating at present.

## 1. Introduction

Acute postoperative pain (APOP) is a common challenge following surgery. Approximately half of all patients report pain of moderate to severe intensity [1]. While some discomfort at the surgical site is expected, excessive pain is far from benign. Higher levels of APOP are associated with a range of complications that can hinder recovery and impose a considerable burden on both patients and healthcare systems [2,3,4].

Acute pain is not merely a sensory experience but a dynamic process shaped by motivational, cognitive, and emotional factors. Several psychobiological models have been proposed to explain how these elements interact in pain perception and persistence. One influential framework is the Motivational–Decision Model [5]. This model postulates that the brain continuously evaluates whether attending to pain is the most adaptive response, balancing competing goals such as escape, rest, or pursuit of reward. Descending modulatory systems, including endogenous opioid-mediated pathways, play a key role in this decision-making process [6]. Closely related are models emphasising fear and avoidance behaviours. The Fear-Avoidance Model suggests that individuals who interpret pain as threatening may develop catastrophic thoughts, leading to avoidance of activity and hypervigilance. This cycle can result in physical deconditioning and emotional distress, ultimately contributing to persistent pain states [7,8]. Meta-analytic evidence confirms that fear of pain predicts pain intensity and disability, although this relationship is influenced by other factors [9].

Other integrative approaches highlight the biopsychosocial context of pain. These models recognise that biological mechanisms interact with psychological factors such as coping strategies and social influences, shaping both acute and persistent pain trajectories [10,11]. Social support, for example, has been shown to buffer pain-related distress and improve outcomes [12].

Together, these frameworks underscore that pain is not a passive sensory event but an active, goal-directed process influenced by motivation, fear, and social context. Understanding these mechanisms is essential for developing individualised interventions targeting both physiological and psychological contributors to pain.

Several reviews have examined predictive coding and Bayesian models of pain, but none have focused specifically on the perioperative setting in the context of individualised care, or provided a concrete expectation-sensitive computational model. This narrative review addresses these gaps by linking Bayesian principles to individualised anaesthesia, analgesia, and future trial design, presenting a preliminary computational model as a suggested strategy for expectation-sensitive clinical trials.

Expectation-modulated pain has been extensively studied in experimental and chronic pain contexts, yet perioperative evidence remains limited. Accordingly, this narrative review does not propose an evidence-based clinical model of expectation-guided anaesthesia. Instead, it offers a conceptual framework that integrates predictive coding principles with emerging perioperative findings to generate hypotheses and identify research priorities. This approach acknowledges current evidence gaps while providing a structured way to understand how expectations may influence APOP. While not sufficient to support guideline changes at present, this framework also points to areas that may inform future perioperative recommendations, including structured expectation assessment, targeted preoperative communication, and expectation-sensitive analgesic planning.

## 2. Methods—Search Strategy

This narrative review was informed by a targeted literature search conducted in PubMed, EMBASE, and CINAHL using combinations of the terms “postoperative pain,” “acute postoperative pain,” “expectations,” “predictive coding,” “Bayesian,” “placebo,” “nocebo,” “perioperative analgesia,” and “individualised anaesthesia”. Search strings included Boolean combinations of these terms. Searches covered literature published between 2000 and 2025, including observational studies, experimental pain studies, interventional trials, and conceptual/theoretical papers relevant to expectation-modulated pain. Exclusion criteria were non-English articles, conference abstracts, and editorials. Reference lists of key articles were screened to identify additional sources.

Because this is a narrative review, study selection was guided by conceptual relevance rather than predefined systematic criteria. Study designs (e.g., experimental, observational, interventional, conceptual) were noted during screening and are described narratively to provide context for the nature and strength of the available evidence. No formal risk-of-bias assessment or evidence-grading framework was applied, as the aim was to synthesise mechanistic and theoretical insights rather than evaluate intervention efficacy. Because the included evidence consists largely of observational studies and small experimental trials, the findings are susceptible to confounding and limited generalisability; these constraints are acknowledged in the narrative synthesis.

## 3. Expecting Pain—A Self-Fulfilling Prophesy

Self-expected pain refers to the level of pain an individual anticipates in a specific context, such as undergoing surgery. These expectations can influence the actual pain experienced postoperatively, reflecting the psychological principle that expectations shape perception, often described as a self-fulfilling prophecy [13]. In perioperative research, expected pain is typically assessed using self-report measures such as a Numeric Rating Scale (NRS) from 0 to 10, or questionnaires including the Pain Self-Efficacy Questionnaire (PSEQ) [14,15], the Expectation for Treatment Scale (ETS) [16] and the Treatment Expectation Questionnaire (TEX-Q) [17].

Preoperative pain self-efficacy is a consistent predictor of postoperative outcomes in observational studies. Higher self-efficacy is associated with lower postoperative analgesic use, including reduced NSAID consumption after total knee arthroplasty [18]. Conversely, spine surgery patients with low PSEQ scores (<22) were more likely to use opioids preoperatively and reported more APOP, disability, fatigue, and depression [19]. These findings support coping confidence as an important determinant of pain trajectories [20] and underpin proposals to include the PSEQ in recent perioperative core outcome sets [21].

Anticipated pain also reliably predicts postoperative pain. In an observational cohort, patients expecting moderate pain (NRS ≥ 4) had higher rates of APOP and greater opioid consumption [22]. Expected pain was the strongest predictor of moderate-to-severe pain after VATS [23], and in a prospective observational cohort of 2258 patients, higher expected-pain categories predicted more intense movement-evoked APOP [24]. Treatment expectations measured by the TEX-Q also influence pain outcomes in observational research, although in some studies they appear to mediate other psychological factors, such as depression [25].

However, recent evidence suggests that explicit expectation ratings may not fully capture the cognitive priors that shape analgesic responses [26]. This experimental study concluded that previous therapeutic experiences, rather than self-reported expectation scores, were stronger predictors of placebo analgesia in both patients with persistent pain syndromes and healthy volunteers. This distinction is highly relevant within a Bayesian framework, where learned priors may exert greater influence on prediction errors than consciously articulated expectations. These findings highlight the need to interpret anticipated pain scores cautiously and suggest that perioperative research should consider both explicit ratings and prior treatment experiences when estimating a patient’s expectations. Beyond observational predictors, early interventional evidence supports the clinical relevance of expectation modulation [27]. Findings from a small interventional trial in trauma surgery patients demonstrated that a structured, expectation-focused educational intervention reduced postoperative opioid consumption while maintaining adequate pain control. This provides a concrete example of how modifying patient expectations, not only pharmacological treatment, can positively shape APOP trajectories. Such findings underscore the potential value of integrating expectation-focused education into preoperative pathways. It is important to note that most perioperative studies examining expectations and postoperative pain are observational, and therefore associations may reflect confounding factors such as anxiety, depression, or pre-existing pain sensitivity rather than causal effects.

## 4. The Bayesian Approach to Perioperative Pain

**Figure 1:**
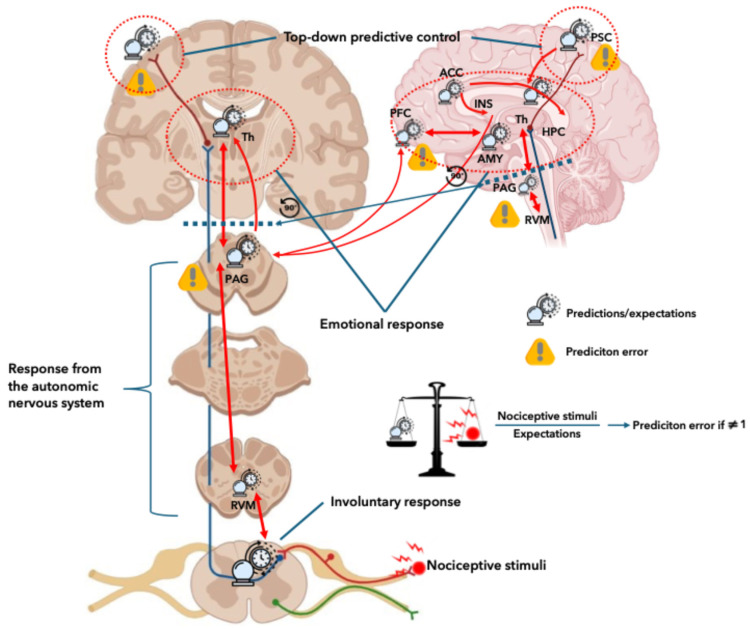
Graphical depiction describing the hierarchal structure, presenting a layered system where pain is processed through prediction and correction at different levels of the nervous system. The top level (cortex) creates a mental model of what pain should feel like based on context and past experiences; A needle prick is predicted as “sharp pain” by the brain before it happens. The middle level (thalamus, brainstem) checks whether incoming noxious stimuli match the brain’s predictions and sends error signals upwards if they don’t; if the prick feels worse than expected, the brainstem signals that this is stronger than predicted. Bottom level (spinal cord) handles raw sensory input and fast reflexes before the brain fully interprets the painful stimulus. Information flows downward as predictions and upward as prediction errors. This constant loop helps the brain fine-tune perception and responses. The scale analogy helps to explain how prediction errors occur; If the scale is balanced, the brain’s model matches, i.e there is little surprise that mandate a response. If the scale is in imbalance something doesn’t fit the model and mandates a response. ACC; Anterior cingulate cortex, AMY; Amygdala, HPC; Hippocampus, INS; Insula, PAG; Periaqueductal grey, PFC; Prefrontal cortex, PSC; Primary sensory cortex, RVM; Rostroventral medulla, Th; Thalamus.

Pain can be understood through a Bayesian framework in which the brain integrates incoming noxious stimuli with prior expectations to create the subjective experience of pain [28]. This model reflects the brain’s constant updating of its predictions about the environment by combining sensory input with beliefs about how painful a stimulus will be (Figure 1). When actual input differs from expectation, a prediction error occurs, prompting behavioural adjustments and updating future expectations [28]. This error is weighted by its precision, which depends on factors like attention, emotion, and pharmacological treatments. High precision means the error strongly influences pain perception; low precision means prior expectations have more influence. The process is organised by a Markov blanket, which separates internal states from external inputs while allowing active inference, adjusting predictions or actions to minimise surprise [29]. This boundary allows the brain to interact with the world without needing to model everything directly. In this way, pain is not a simple reflection of tissue damage but a dynamic negotiation between expectation and sensation, explaining why context and analgesia can profoundly alter the subjective experience of pain [29].

Expectations may therefore be powerful modulators of perceived pain and treatment response. A clear clinical example is placebo analgesia, demonstrated in an individual-participant meta-analysis, which can reduce reported pain without altering the brain’s nociceptive signature [30]. In an experimental trial, healthy volunteers who received placebo treatment after a painful stimulus and held more confident expectations and experienced greater reductions in reported pain [31]. Functional MRI revealed that the periaqueductal gray (PAG) and rostral ventromedial medulla (RVM) encode this precision signal [31]. In a small clinical experimental study (open-hidden analgesia paradigm), patients undergoing thoracotomy received buprenorphine 3 days postoperatively, along with an infusion of normal saline [32]. Patients were divided into three groups, where the first group did not receive any information about the infusion (natural history group), whereas the second group was told that the saline infusion either contained a strong analgesic or placebo (double-blinded natural history group). The third group was told that the saline infusion contained a strong analgesic (deceptive group). All three groups recorded similar analgesia scores but with lower opioid requirements in the deceptive compared to the natural history groups and in double-blinded compared to the natural history group. This suggests that expectation alone can drive meaningful placebo analgesic effects. Conversely, the nocebo effect is a phenomenon where negative expectations or beliefs about a treatment lead to worsening symptoms or adverse effects, even when the treatment itself is inert or harmless. Some evidence from experimental studies suggests that the nocebo effect might have a greater impact on outcomes than placebo [33]. Evidence in the perioperative setting remains limited, though emerging research suggests this may change soon [34].

**Figure 2:**
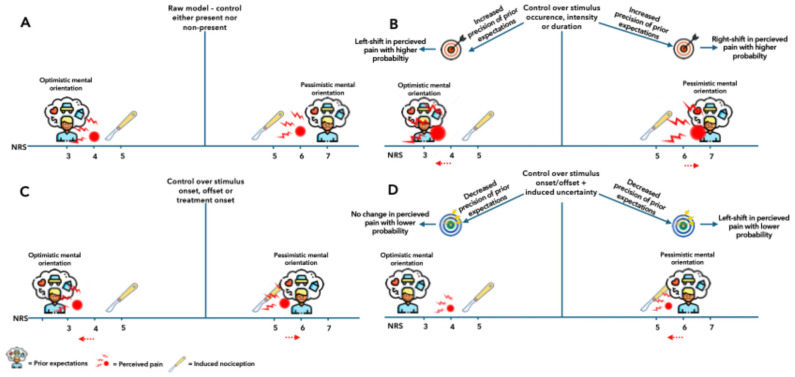
Conceptual Bayesian model illustrating how expectations shape perceived postoperative pain. (**A**). Optimistic expectations reduce the subjective weighting of nociceptive input, whereas pessimistic expectations increase it. (**B**). Greater perceived control increases the precision of expectations, making them more influential in shaping pain perception. (**C**). Predictable timing of stimuli or treatments can shift expectations and perceived pain toward lower or higher intensities depending on orientation (optimistic vs. pessimistic). (**D**). When temporal uncertainty reduces expectation precision, perception relies more heavily on raw sensory input. These panels illustrate conceptual mechanisms rather than quantitative effects.

Predictive coding suggests that when people expect a mild stimulus (e.g., NRS 3—optimism) but experience something more intense (e.g., NRS 5), their perceived pain often falls between the two (e.g., around NRS 4) (Figure 2A). Conversely, if they expect severe pain (e.g., NRS 7—pessimism), the same stimulus (e.g., NRS 5) may feel worse (e.g., NRS 6) (Figure 2A). This illustrates how expectations shape perception.

Control is potentially a powerful modulator because it influences expectations. Patients using patient-controlled analgesia often report lower pain scores [35]. However, in scientific literature, the definition of control varies, such as over stimulus timing, intensity, or duration [36]. Within a Bayesian framework, control works through two mechanisms: it increases the precision of prior expectations and shifts those expectations towards lower pain (Figure 2B). Both alter how the brain integrates sensory input and expectation.

Clinically, a patient with a traumatic past surgical experience may expect severe pain and feel confident in that belief. Even if the procedure is less painful, their perception may be amplified. This mechanism may help explain why self-reported pain expectations are associated with APOP [13,14,15].

Having control over when pain starts or when treatment begins, such as being able to alleviate pain or initiate analgesia, can reduce perceived pain by shifting expectations towards lower intensities (Figure 2C). For example, a diabetic patient self-administering insulin often reports less pain than if the injection is given by someone else [37].

Similarly, initiating a painful stimulus (e.g., pressing a button) may enhance the sense of control and may lower pain. However, if there is a delay between the action and the onset of pain, this benefit diminishes. This delay introduces temporal uncertainty, making the brain less confident in its prediction and more reliant on raw sensory input. Predictable stimuli amplify the effect of optimistic expectations, whereas unpredictability reduces it (Figure 2D). Interestingly, when expectations are pessimistic, uncertainty may dampen perceived pain because the brain discounts imprecise prior expectations (Figure 2D).

In summary, pain perception reflects an integration of expectations and sensory input, updated when mismatches occur. While this Bayesian model is well studied in persistent pain syndromes [36], it is hypothesised that this framework can also be applied in the perioperative setting and in APOP, where patients often have little control.

## 5. Framing the Bayesian Model of Postoperative Pain in Clinical Trials

Predictive coding frames perception as an active, dynamic concept in which prior expectations interact with incoming nociceptive signals to shape the final experience of pain. The influence of these expectations depends on their precision, which is modulated by factors such as control and uncertainty.

A small experimental psychobiological study (n = 45) explored how different models of pain expectations affect subjective pain perception and examined individual variability in expectation sensitivity [38]. Participants viewed cues (pie charts or playing cards) indicating probabilities of receiving specific pain intensities, then received a mild electric shock and rated their pain. The analysis tested six models, each increasingly complex, to capture how participants’ expectations influenced their pain experience:

1. Baseline model assumptions: Pain perception depends only on the actual noxious stimulus. This model acted as a reference point to be able to see if adding expectations improved predictions.
2. Multimodal prior model assumptions: Pain perception depends on the intensity of noxious stimuli + information about the cue. This assumes that people form expectations based on all possible intensities of the noxious stimuli, shown in the cue.
3. Mean-only model assumptions: Pain perception depends on the intensity of noxious stimuli + information about the mean value of the cue. This assumes that people simplify the cue and expect an average pain intensity. For example, if the cue states that it is 50% chance of NRS 4 and 50% chance of NRS 7, the expected pain is NRS 5–6.
4. Mean and variance model assumptions: Pain perception depends on the intensity of noxious stimuli + the mean and variance of the cue. This basically assumes that people consider both the average pain intensity of the cue (as in mean-only model) but also its variance. If the cue states that there is a 25% chance of NRS 3 and 75% chance of NRS 7, uncertainty is introduced leading to a reduced influence of the expectations (less control = less precision).
5. Full model assumptions: Pain perception depends on the intensity of noxious stimuli + independent cue information + trait-like features (pessimism vs. optimism). This assumes that someone who always expects high pain, regardless of the cue, will perceive pain more intensely.
6. Hierarchical model assumptions: Pain perception depends on the intensity of noxious stimuli + cue information (average pain + uncertainty) + trait-like features. This assumes real-life scenarios where people are different: one may be sensitive to pain cues, another may have a strong pessimistic bias, and a third may be less influenced by expectations altogether. Thus, these variables were allowed to vary freely.

Expectations exerted a stronger influence on perceived pain than the baseline model, but this effect diminished when uncertainty was introduced. Stable personality traits, such as dispositional optimism or pessimism, also shaped pain perception. The hierarchical model best captured individual differences in expectation sensitivity. Although limited by sample size and uncertain generalisability to perioperative settings, these findings highlight the relevance of psychological preparation before surgery. Expectations may either support or hinder recovery, depending on how they are shaped preoperatively [38].

Predictive coding of nociceptive input thus offers a promising framework for identifying individuals who are particularly sensitive to expectation effects. This can inform individualised preoperative communication and the development of quantitative trial designs. Future clinical trials should explicitly incorporate expectation modelling, for example by stratifying participants into high vs. low expectation-sensitive groups, or by using statistical adjustment for individual expectation profiles to isolate the true effect of perioperative interventions. In addition, trials should test targeted expectation-modulating strategies (e.g., structured education, uncertainty-reduction protocols, or control-enhancing measures) to determine whether modifying prior expectations can systematically improve postoperative outcomes.

Unfortunately, clinical trials in this field have not yet provided the high-quality, adequately powered evidence needed to determine whether modifying expectations can meaningfully improve postoperative outcomes. However, some trials have started to include patients to see if impacting preoperative expectations can improve postoperative outcomes [39].

A more focused future research agenda should prioritise designing trials that experimentally manipulate expectations or include expectation phenotyping as a core methodological component, ensuring that prior expectations are fully understood and accounted for when evaluating analgesic strategies. This will clarify whether current perioperative practices are unintentionally setting patients up for success, or for failure.

Although this review presents a conceptual framework, it is still possible to outline how a Bayesian approach could be applied in principle and how it might inform perioperative care as a computational model. As a core concept, integration includes priors (e.g., patient expectations and psychological factors), likelihood (e.g., biological and procedural determinants of nociception), posterior (e.g., predicted postoperative pain) and prediction errors (e.g., mismatch between predicted and observed pain).

Step 1: Prior expectations can be estimated using brief tools such as anticipated pain scales (NRS), pain self-efficacy scales (PSEQ), trait expectation measures (e.g., TEX-Q), together with previous surgical experiences. Expectation measures may be standardised and combined into a composite expectation prior using a weighted sum, with weights derived either a priori (equal weighting) or empirically (e.g., from regression coefficients predicting postoperative pain).

Step 2: The likelihood, representing the data-driven expected nociceptive input given the surgical and biological context, could be approximated using routine perioperative variables including surgical invasiveness, comorbidities, individual risk factors (e.g., gender, age, preoperative pain and opioid tolerance), analgesic technique (e.g., regional vs. systemic) and intraoperative nociception indices. In such models, β coefficients for each co-factor are empirically derived using regression or hierarchical modelling, with each β multiplied by the corresponding input variable (coded nominal, dichotomous or continuous in nature). However, there is currently no consensus on which factors are most important [40].

Step 3: The variables in step 1 (expectations) and step 2 (nociceptive load) can then be integrated to estimate a posterior prediction of postoperative pain or APOP risk. One pragmatic approach is first to fit two separate models predicting postoperative pain: one using expectation measures only and one using nociceptive risk factors only. The residual variance from each model is calculated using standard regression methods, and precision is defined as the inverse of this residual variance (precision = 1/variance). These precision values can then be normalised to yield weights for expectations and nociceptive load, which are used to generate an individualised posterior prediction of postoperative pain by integrating expectation-related priors with procedural and non-procedural likelihood factors. This posterior estimate has the potential to be used to stratify patients preoperatively, guide individualised analgesic strategies, and serve as a baseline risk variable in clinical trial designs.

Step 4: Prediction error can be calculated as: Prediction error = Observed pain − Predicted pain. Large positive prediction errors can be interpreted as APOP being worse than the model predicted, whereas large negative prediction errors indicate APOP being better than predicted. At the individual level, large prediction errors may potentially flag patients for intensified multimodal analgesia or targeted expectation-focused communication. At the model level, systematic prediction errors can be used to update weights (e.g., the precision assigned to priors and likelihood) and refine which variables are most informative.

## 6. Expecting Pain in Individualised Anaesthesia and Analgesia—The Discussion

Applying predictive coding and Bayesian models of APOP to perioperative medicine offers a useful framework for understanding why patients respond differently to the same surgical stimulus or analgesic regimen. In this view, pain is not solely a consequence of nociception but reflects how the brain interprets sensory input in light of prior expectations and the confidence placed in them (their precision) [28]. This helps explain why context, communication, anxiety, attention, and previous experiences may play a meaningful role in postoperative pain.

During anaesthesia, the brain’s ability to update expectations, i.e., to correct prediction errors, may be reduced [41]. Bayesian principles therefore offer a way to think about more individualised anaesthetic care. Adaptive anaesthetic and analgesic dosing strategies might, in principle, draw on real-time physiological markers of nociception (e.g., real-time intraoperative nociception monitoring, markers of sympathetic tone) together with known patient characteristics to better understand the balance between nociceptive input and prior expectations. However, this remains a theoretical possibility, and its clinical feasibility has yet to be established. When expectations cannot be modified during anaesthesia, clinicians may still aim to minimise prediction errors by maintaining consistent analgesic depth and avoiding large fluctuations in nociceptive stimuli. This is particularly relevant for patients who enter surgery with high anxiety or strong expectations of severe pain, although this remains to be empirically tested.

Individualised analgesia could also target both bottom-up and top-down contributors to pain. Bottom-up strategies target the nociceptive system directly, such as enhancing descending inhibition or reducing peripheral and central sensitisation. Top-down strategies influence how pain signals are interpreted. Interventions that strengthen positive expectations, such as structured preoperative communication or conditioning-based approaches, may increase the precision of analgesic expectations and potentially enhance the effect of pharmacological treatments and reduce opioid requirements [42]. Patients with high anxiety may assign excessive precision to nociceptive prediction errors, as suggested by experimental and observational studies, interpreting signals as more threatening and being less reassured by verbal information [43,44]. For such individuals, expectation-focused communication may need to be combined with robust analgesic strategies to reduce pain amplification driven by hypervigilance.

The computational model proposed in this narrative review illustrates how expectation-related measures and procedural and non-procedural determinants of nociceptive load could be integrated to generate an individualised posterior estimate of postoperative pain risk. Such an estimate could support preoperative stratification, helping identify patients who may benefit from enhanced multimodal analgesia, targeted expectation-modifying communication, or closer postoperative monitoring. The model also provides a structured way to quantify prediction error, the mismatch between predicted and observed pain, which may help clinicians detect patients whose recovery deviates from expected trajectories. Beyond individual care, this framework could inform future clinical trials by enabling expectation-based stratification, improving baseline risk adjustment, and testing whether interventions that modify expectations meaningfully shift predicted pain trajectories.

It is important to emphasise that the Bayesian and predictive coding perspectives presented here constitute a conceptual framework rather than a validated clinical model. Although these theories are well supported in experimental pain research, direct perioperative evidence remains limited, and the framework is therefore intended to guide future hypothesis-driven clinical studies rather than immediate changes to anaesthetic practice.

A related consideration is the distinction between statistical significance and clinical utility. Several perioperative studies report statistically significant associations between preoperative expectations and postoperative outcomes, yet such findings do not necessarily imply meaningful differences in analgesic requirements, postoperative pain or recovery trajectories in real-world settings. The available evidence is constrained by the predominance of observational cohorts and small experimental studies, many from single-centre settings, which limits causal inference, increases susceptibility to confounding, and reduces generalisability across surgical populations. Furthermore, experimental pain paradigms offer mechanistic insight but may not reflect the complexity of perioperative nociception or the influence of anaesthesia. As a result, the practical relevance of expectation-modulated effects remains uncertain and requires confirmation in adequately powered perioperative trials designed to test whether modifying expectations produces measurable improvements in perioperative outcomes.

Finally, expectations operate alongside well-established procedural and non-procedural risk factors for postoperative pain. Surgical invasiveness, tissue injury, inflammatory responses, pre-existing pain, comorbidities, demographic factors, psychological strain, genetic pain sensitivity, and the choice of perioperative analgesic techniques all contribute to nociceptive input. Within a Bayesian framework, these factors shape the “likelihood” term, the sensory evidence against which expectations are compared. Expectations do not replace biological determinants but interact with them, influencing how strongly nociceptive signals are weighted and interpreted. Integrating both psychological and biological contributors provides a more balanced and realistic conceptual model of postoperative pain, while acknowledging that the relative influence of each component remains to be empirically quantified.

## 7. Conclusions

### 7.1. Clinical Summary

Patient expectations are recognised modulators of pain perception and may influence APOP, although the strength of this effect in perioperative settings remains uncertain. High anticipated pain, low self-efficacy, uncertainty, and reduced sense of control have the potential to amplify pain, whereas structured communication, predictable care, and confidence-enhancing interactions may decrease pain intensity and reduce opioid requirements. Integrating these factors into routine preoperative assessment may offer a practical pathway toward more individualised anaesthesia and analgesia.

### 7.2. Future Directions

Future research should prioritise distinguishing explicit expectations from learned priors through validated psychometric and computational approaches; evaluating expectation-modulating interventions, such as structured communication, educational programmes, and strategies that enhance predictability or control, in perioperative clinical trials; incorporating expectation measures into preoperative risk stratification; and exploring how anaesthetic agents influence prediction-error signalling. These steps may support the development of a more precise and clinically actionable model of expectation-sensitive postoperative pain management.

Future clinical trials are required to determine whether expectation-modulating strategies can be systematically integrated into individualised anaesthesia and analgesia. Until such evidence emerges, the Bayesian and predictive coding perspectives outlined here should be viewed as hypothesis-generating tools rather than evidence-based clinical recommendations.

## Data Availability

No new data were created or analyzed in this study. Data sharing is not applicable to this article.

## Abbreviations

ACC	Anterior cingulate cortex
AMY	Amygdala
APOP	Acute postoperative pain
ETS	Expectation for Treatment Scale
HPC	Hippocampus
INS	Insula
NRS	Numeric rating scale
PSEQ	Pain Self-Efficacy Questionnaire
PAG	Periaqueductal grey
PFC	Prefrontal cortex
PSC	Primary sensory cortex
RVM	Rostral ventromedial medulla
TEX-Q	Treatment Expectation Questionnaire
